# The Fitting and Furnishing of Wards and Operating Theatres

**Published:** 1906-09-08

**Authors:** 


### THE FITTING AND FURNISHING OF WARDS AND OPERATING THEATRES.

In the following articles we propose to discuss the modern methods of fitting and furnishing hospital wards, corridors, operating theatres, post-mortem rooms, and mortuaries, having regard to modern requirements.

### Floors.

The floors of every modern public building, in fact of every building of any size, are obliged to conform to very much more severe requirements than were ever dreamed of in old-time buildings. In old buildings, wooden joists were laid across the space to be floored; and wooden planks were nailed or screwed to the joists, and as long as the floor, with its supports, was sufficiently strong to carry the weights that were placed upon it, and the wood of which the floor was composed was sufficiently seasoned and carefully fitted, to prevent the devolpment of holes between the boards, and in the boards, that was all that was required. In these days, however, the floor is required to be sound proof if possible, and, as far as possible, certainly to be water proof in the fullest sense of the term, and to be fire-proof as far as it is possible to make any substance, or built-up material. Put in another way, where with old-time houses, except in some very old houses, where there were large spaces in the floors for other purposes, concealment and things of that kind, very little attempt was made to prevent the transmission of sound, or the spread of fire; in the modern building every effort is made to interpose as much resistance as possible between the rooms occupying different floors for sound and for heat. In addition to this, in the modern hospital, aseptic requirements demand that the floor shall present no harbour, under any of the conditions that may rule, for pathogenic germs. To comply with this requirement, which is practically overshadowing all the others, the floor must be perfectly smooth at all times. There must be no corners where it joins the walls, and there must be no cracks of any kind, such as there were in old wooden floors. It goes without saying, also, that every part of the floor must be easily accessible for thoroughly cleansing, and must be able to be cleaned with, if necessary, aseptic substances. On the other hand, certain other requirements of the floors of wards and operating theatres can hardly be quite lost sight of. Nurses have to be on their feet for a great number of hours, and have to walk what would amount to a great distance, in their continual passages through the ward. Surgeons also have to stand for long periods by the side of the operating table, or the bedsides of the patients. For these reasons a floor which is very hard, and devoid of resilience, is not so good, whatever may be its aseptic properties, as one that has a little spring. The latter is not so tiring to the feet of nurses and surgeons. Again, some floors which are perfectly aseptic when intact are cold comparatively; that is to say, they require a considerable amount of heat to raise their temperature, and they also very largely increase the amount of breakages of crockery, glass, etc.

The above requirements are met in the modern hospital in several ways. There is the floor made of blocks of hard wood, set on edge, in what is known as the herring-bone fashion, the blocks being laid either on a cement sub-floor or in pitch. There are also the numerous mosaics, of which the "Terrazzo" is the best known, in which small pieces of marble, granite, or vitreous material are embedded in a sub-floor of cement, pressed down, and the upper surface ground smooth. Then there are the numerous substances designed to take the place of the mosaics, in which sawdust and other materials, variously named by their manufacturers as vegetable and mineral, are ground to a powder, mixed with various kinds of cement, laid on a sub-floor of cement, in a plastic condition, rolled, and allowed to dry. And, lastly, there are the linoleums, which are finding considerable favour. As usual in practical engineering work, there is no absolute rule. There is no best material. All of the above have their good points, all have their disadvantages, and all are more suitable for some cases than for others. It should be mentioned that the substances described only form the surface or skin; that is to say, a comparatively small portion of the total thickness of the floor. The modern floor extends the whole depth of the steel joist which supports it, and sometimes a little below. It follows, therefore, that a good deal of the trouble which has arisen with some forms of flooring, the mosaics, for instance, which have been found to crack very much, and particularly over the steel joists, is really due to the way in which the floor proper has been built up, and in some cases to the way in which the whole building is erected. Foundations, for instance, are not always as perfect as could be wished, and a slight settlement in one part of a building may easily lead to the cracking of what is virtually the skin of the floor. It is very doubtful whether the cracks in "Terrazzo" that are, unfortunately, so frequent, are due to the expansion of the girders by heat. It is more probable that they are due to unequal settling of parts of the floor or to movements of the building itself. Nevertheless, cracks in the mosaics are unfortunately frequent. Cracks in the made-up plastic materials, of which "Euboelith" and "Segalith" are well-known examples, are less than with the mosaics. Hard wood blocks, carefully laid with a good sub-floor, and well-beeswaxed on the surface, the minute interstices between the blocks being carefully filled with the beeswax, and kept filled, appear to answer as well as anything. In this connection the writer would call attention to the new Australian woods, Karri and Jarrah, which are very hard, and are claimed to be non-inflammable and aseptic in themselves. By the recently introduced process known as Haskinsing, any wood can be made aseptic. The troubles with the mosaic floors, and doubts as to the aseptic properties of wood blocks, have led to the adoption of a special form of linoleum, which so far appears also to answer very well indeed. It is a *sine qua non* that the pattern of the linoleum shall extend right through the whole of the substance, so that it shall wear down quite evenly. This linoleum is described as "inlay." The linoleum must also be of a good thickness. The floor on which it is laid is made thoroughly aseptic before laying, and the linoleum is laid either on a cement sub-floor, or it can be pasted to a wooden floor with a strong paste, and the rubber solution at the joints. The substance must be absolutely dust and germ-proof, and it must be capable of being washed, scrubbed, and treated with antiseptics, just as the harder wood or mosaic floor would be. It has the great advantage of being elastic, warm, and comfortable to the feet, as well as pleasant to the eye.

